# Evaluating the detection of small brain lesions in magnetic resonance using deep learning

**DOI:** 10.3389/fnins.2026.1833713

**Published:** 2026-07-13

**Authors:** Alberto Nogales, Miguel Ángel Sicilia, Carolina de la Pinta, Elena García-Barriocanal, Álvaro J. García-Tejedor, Diego Guadalupe

**Affiliations:** 1 CEIEC, Research Institute, Universidad Francisco de Vitoria, Pozuelo de Alarcón, Spain; 2 University of Alcalá (UAH) Computer Science Department, Alcalá de Henares, Spain; 3 Instituto Ramón y Cajal para la Investigación en Salud (IRYCIS), Biomedical Data Science and Engineering Group, IRYCIS, Madrid, Spain; 4 Radiation Oncology Department, Ramón y Cajal Hospital, Biomarkers and Therapeutic Targets Group, IRYCIS, Madrid, Spain

**Keywords:** artificial intelligence, deep learning, magnetic resonance, small brain tumour, U-net

## Abstract

**Introduction:**

Detecting brain lesions using Artificial Intelligence methods has been a focus of prior research, with numerous datasets supporting this task to improve clinical results. However, evaluation metrics and reported results often summarise overall performance without considering variations in lesion size. In clinical practice, the detection of small tumours is particularly critical for early diagnosis and treatment effectiveness.

**Methods:**

This study evaluates the performance of Artificial Intelligence models on established datasets with a specific focus on lesion identification and lesion size. We introduce a novel Deep Learning model tailored to detect small brain tumours in Magnetic Resonance Imaging, integrating a clinically defined “small tumour” concept into both the training and evaluation processes.

**Results:**

The proposed approach demonstrates robust performance, achieving loss values ranging from 1.5 to 11.1 and Dice Scores between 96.3% and 98.1% across multiple datasets.

**Discussion:**

The main contribution of this work is the incorporation of clinically meaningful lesion-size information into model development and assessment. These findings suggest that explicitly considering small tumours can improve the clinical relevance of Artificial Intelligence systems for brain lesion detection and support earlier diagnosis and more effective treatment planning.

## 1 Introduction

The standard treatment of primary brain tumours (benign or malignant) or metastatic brain tumours includes surgery, radiotherapy, and systemic therapy (chemotherapy, hormonotherapy, etc.), depending on histology, size, location, and relationship with brain structures (Rudà et al., 2022; Vogelbaum et al., 2022; Horbinski et al., 2023). When lesions are detected with large volumes or near critical structures, they limit surgical and radiosurgical options, affecting therapeutic outcomes and negatively impacting neurocognitive functions and, therefore, quality of life. Early detection also allows a correct diagnosis and staging of patients, which enables a more appropriate treatment or even a change of treatment in those cases in which it has already begun.

One of the most crucial steps in the staging and follow-up of brain cancer patients is the study of the central nervous system by Magnetic Resonance Imaging (MRI). These studies are of enormous importance, both for screening patients with unknown brain lesions and for the follow-up of patients with brain tumours who have already been treated, as they may develop recurrences or new lesions (mainly in metastatic disease). MRI is a technique that produces detailed images of organs and internal tissues using magnetic fields, large magnets, and radiofrequency waves, combined with a computer system (Hernández et al., 2009). This is then used as a functional MRI, a non-invasive and sensitive tool to map brain activity in humans (Moraes et al., 2019). Apart from that, it has other benefits, such as having a better contrast compared with other medical tests or multi-planar images that help in studying the extension of tumours.

However, even with MRI scans, certain cases remain difficult to detect, even for expert neuroradiologists. This is the case of patients with multiple metastases or small brain tumours (meningioma, neurinoma, etc), those with a diameter smaller than 1 cm according to Moraes et al. (2019), Grishchuk et al. (2023), or Bhimani et al. (2022). The size choice is clinically motivated rather than statistical: sub-centimetre lesions mark a recognised management threshold in neuro-oncology. For example, Moraes et al. (2019) show that treatment outcomes are strongly size-dependent in this range, setting the smaller size to 1 cm. In Grishchuk et al. (2023), dedicated stereotactic-radiosurgery guidelines treat brain metastases ≤1 cm as small with specific dose and planning considerations. In pituitary tumours, the <1 cm (microadenoma) boundary is a standard sizing criterion that influences the indication for surgery (Bhimani et al., 2022). Adopting this threshold therefore aligns the detection task with a decision boundary that matters in clinical practice. Although the 1 cm criterion is applied uniformly across the meningioma, glioma, pituitary, and metastatic lesions considered here, its precise clinical implications and the appearance of sub-centimetre lesions can vary with tumour type and with MRI acquisition protocol (sequence, contrast, slice thickness, and resolution). The problem of recognizing small tumors is that this task is made visually and depends heavily on the physician’s expertise, who may need to dedicate more or less amount of time. This dependency leads to high variability in diagnosis between individual experts and across different experts (Jiang et al., 2023). At this point, Artificial Intelligence techniques can be used as a way to aid the experts. This task can be encompassed as an object detection task in the field of computer vision.

The main motivation for the present work lies in using Deep Learning (DL) models to detect primary small brain tumours. As far as we know, there is only one work (Ngo et al., 2020), that addresses this problem, but it arguably uses a concept of small brain tumours different from our interpretation here. Further, the model is only trained with 3-dimensional MRIs, and here we approach the task with both 3-dimensional and 2-dimensional MRIs, obtaining better results than the state of the art.

This work should be considered a first approach to exploring the benefits of DL for the segmentation of small brain tumours in MRI. Although the DL techniques and mechanisms employed have been previously proposed, the main novelty of this work lies in the integration of a clinically grounded definition of “small tumours” (diameter < 1 cm) into the entire model development and evaluation process, addressing a gap overlooked by previous studies that rely on arbitrary statistical thresholds that may introduce biases. This medical perspective, combined with a thorough quantitative and qualitative analysis, positions our work as a practical and expert-validated approach to early brain lesion detection. In addition, we provide an evaluation across both 2D and 3D MRIs, a capability not systematically demonstrated in previous literature. Furthermore, we include a medical expert evaluation of diagnostic errors, considering oedema, vascularisation, and tumour location, aspects often missing in prior technical studies. This helps bridge the gap between the performance of AI techniques and their clinical relevance. In summary, unlike previous studies, this work specifically addresses clinically defined small brain tumours (<1 cm) using both 2D and 3D MRIs, combining attention-guided segmentation with expert clinical validation.

The contribution of the paper is, first, exploring the benefits of DL techniques for the task of detecting small brain tumours in MRIs, but using the medical concept of those having a diameter smaller than 1 centimetre. Another interesting point is that this model can be used with 2-dimensional and 3-dimensional MRIs. Finally, we provide a medical evaluation that could point out the weaknesses of the model, so that particular improvements could be made in future works in the field of computer science.

The rest of the paper is structured as follows. Section 2 provides a comprehensive overview of the current state of AI and its applications in brain tumour detection. Section 3 outlines the datasets used and the various methodologies employed. Section 4 described the proposed solution for the particular use case of small brain tumours, presenting the results obtained and the performance evaluation of the most effective model following human assessment. In Section 5, the discussion about the results is presented. Then, all the limitations of the work are compiled in Section 6. Lastly, Section 7 presents concluding remarks and highlights some future work.

## 2 Related work

As mentioned above, this work is aimed at detecting small brain tumors in MRIs using Deep Learning models. In this section, we are describing the state of the art, showing different works that go from the application of classical Machine Learning techniques in brain tumour detection or classification to the particular case of detecting those with small sizes using DL models and MRIs. For example, Amin et al. (2022) surveys ML techniques for the classification and detection of brain tumours. More specialised is the work of Soomro et al. (2023), which is focused on the use of MRIs for brain tumour detection using ML techniques.

When employing classical ML techniques, several interesting approaches emerge. For instance, algorithms such as Support Vector Machine (SVM), Random Forest (RF), K-Nearest Neighbour (KNN), and Linear Discriminant Analysis (LDA) were used for the classification of tumours based on image features. Similarly, Vijithananda et al. (2022) extracted from MRI tumour features like mean pixel values, skewness, and kurtosis, employing various ML classification algorithms. In the study by Zacharaki et al. (2009), focusing on meningiomas, they extract other features such as regions of interest and cell contours for classification using SVM and Local Binary Pattern (LBP). Furthermore, Podnar et al. (2019) explores alternative tumour diagnosis methods, employing blood analysis coupled with ML models. Notably, while these studies are relevant to the project, none specifically address the challenge of detecting small tumours by applying DL models.

A similar DL to the one proposed in this paper has proven effective in other medical-imaging detection tasks, such as breast cancer detection in mammography (Altameem et al., 2022). DL has also been applied to other neuroimaging detection problems using architectural ingredients close to ours. For example, Mahanty et al. (2024) attention (squeeze-and-excitation) and multi-scale feature extraction with dilated convolutions and feature-pyramid networks have been used to detect very small regions in brain MRI for Alzheimer’s disease classification. In the case of DL for tumor classification, different works have been proposed according to this systematic review (Rasool and Bhat, 2024). However, it only mentions two papers about small brain tumours, but they are not targeted only at working with this particular size. An interesting work is presented by Noreen et al. (2020), where two methods are applied; the first extracts MRI features using Inception-V3 and DenseNet, and the second classifies them using a SoftMax layer. The use of a hybrid model called GoogLeNet, which is based on a CNN, is proposed by Raza et al. (2022) for the classification of gliomas, meningiomas, and pituitary tumours. Another interesting work is Aamir et al. (2022), where MRIs are pre-processed to improve visual quality before applying two pre-trained DL models for feature extraction. The resulting feature vectors are combined using the partial least squares (PLS) method, and top tumour locations are identified through agglomerative clustering. Finally, Khan et al. (2020) applies some preprocessing techniques with pre-trained VGG16 and VGG19 for feature extraction. Next, these characteristics are obtained with an extreme learning machine (ELM) for feature selection and the final classification. Although these works use brain tumour MRIs to train DL models, they solve classification problems. In our case, the purpose is to detect the tumours, but those that are considered to be of small size.

Previous work studying the problem of brain tumour detection using MRIs with DL models is described in what follows. In Havaei et al. (2017), it segments brain tumours in multimodal MRI images by applying a cascaded CNN together with a precision mechanism called Distance-Wise Attention (DWA), which is presented as the great novelty of the work. The problem of tumour size is addressed by analysing each slice, observing how the size of the tumour progressively varies from the time it appears until it reaches its maximum and subsequently decreases. In Díaz-Pernas et al. (2021), the most innovative point consists of preprocessing MRIs by applying three spatial scales and CNNs as DL techniques. In this case, tumour size does not have a specific range but includes different spatial views such as sagittal, coronal, and axial. Another interesting work is Alwadee et al. (2025) which introduces a lightweight 3D Attention U-Net with parallel convolutions to segment brain tumors effectively while drastically reducing computational costs. It is designed to be deployable in resource-limited settings by balancing multi-scale feature extraction with attention mechanisms. Then, Cheng et al. (2025) proposes a coarse-to-fine multi-modal brain tumor segmentation framework that uses cross-attention fusion, multi-scale context perception, and feature integration to accurately delineate lesions. Finally, Sadad et al. (2021) obtain brain tumour MRI features by using ResNet50 and then apply evolutionary algorithms and reinforcement learning. Models are trained with small tumours of meningioma, glioma, and pituitary tumours, but also tumours of greater size. As can be seen in all these papers, Deep Learning models are applied to MRI with brain tumors, but none of them specifically address small-sized tumors. Although the previous works perform brain tumor segmentation regardless of tumor size, their performance ranges from 82.8 to 92.0 DCS, which is at least 4% lower than that of our model. Only the last work performs better in terms of segmentation performance, improving on our model by 1 point; however, as we mentioned before, these models are not specifically focused on segmenting small brain tumors.

As far as we know, the only work applied to small brain tumours is by Ngo et al. (2020) using U-Nets for the segmentation of tumours in 3D MRIs. The difference between their concept of small brain tumours and the one adopted here is that it depends directly on the size of the images and not on a medical criterion. By using the clinical concept of considering a small tumour, those having a diameter below 1 cm, we avoid clinically inappropriate biases. Also, we can work with both 2D and 3D MRIs, which makes the solution generalizable no matter the dataset used. Apart from that, we have provided a clinical expert evaluation of error types, analysing the impact of vascularisation, location, and edema, which offers insights that go beyond segmentation metrics. In terms of performance, we report an improvement of about 7% in a similar experiment.

## 3 Materials and methods

In this section, the datasets, methods, architectures, and metrics selected are described.

### 3.1 Datasets used

Due to the scarcity of datasets that meet our needs, we found it necessary to use publicly available datasets. The following are the datasets found in our literature analysis that were considered as candidates.

Brain Tumor Segmentation (BraTS).^1^ This is a well-known dataset as it is part of a public challenge for brain segmentation, so different versions can be found. The used version corresponds to 2021. This version includes 367 3D MRI scans with small tumours. It is the second dataset with a greater number of small tumours of low-grade and high-grade gliomas (Baid et al., 2021). Its main disadvantage is that the MRI scans are in 3D, which are much more expensive to obtain than 2D and, therefore, more complex to process by DL models.

The Cancer Genome Atlas Low-Grade Glioma (TCGA-LGG).^2^ Dataset with 2D MRI scans, which contains 335 small tumours of Low-Grade Glioma images (Pedano et al., 2016). However, a large part of the dataset has no tumours in its MRI scans.

Brain T1-weighted CE-MRI dataset.^3^ Dataset with 2D MRI scans having 1,318 cases with small tumours of meningioma, glioma, and pituitary tumours. Because it is 2D and contains the highest number of MRI scans with small tumours seems to be the most suitable one. This data was compiled and obtained by Nanfang Hospital and General Hospital, Tianjing Medical University of China (Cheng et al., 2015).

LGG-1p19qDeletion.^4^ Dataset with 3D MRI scans with 23 small tumours of Glioma Grade 2 and 3 (Erickson et al., 2017). The number of MRIs with small brain tumours makes this dataset not very good for object detection tasks.

In Table 1, all the datasets are described in depth, showing different features. The differentiation of small brain tumours has been made taking into account our medical criteria of less than 1 cm. At first glance, it appears that all datasets can be used except LGG-1p19qDeletion, which contains only a limited number of MRI scans with small brain tumours.

**Table 1:** In-depth description of open datasets.

Dataset name	Type of tumours	Size	Small tumours	Tumors of greater sizes	Type of MRIs
BraTS 2021	1,251 MRIs with low-grade and high-grade gliomas.	240 × 240 × 155	367	884	T1, T2, T1-CE and Flair, all in 3D
TCGA-LGG	3,929 MRIs, only 1,373 with low-grade gliomas, the rest have no tumours.	256 × 256	355	1,038	T1W in 2D
Brain T1-weighed CE-MRI	3,064 MRI scans, which are divided into 708 meningiomas, 1,426 gliomas, and 930 pituitary tumours	256 × 256	1,318	1,746	T1-CE in 2D
LGG-1p19qDeletion	159 patients with grade 2 and 3 glioma	25 × 6256 × 20:60	23	139	T1W and T2W in 3D

Although the significant differences between datasets pose a problem for generalization, we intentionally retained this heterogeneity to ensure that our findings remain applicable to the diverse clinical settings in which small brain tumours are diagnosed in the real world. An important challenge in this way is class imbalance. This imbalance appears both at the image level and pixel level, since tumour regions occupy a substantially smaller area than healthy tissue, particularly in the case of small lesions. The problem is more pronounced in datasets such as TCGA-LGG, where a large proportion of MRIs do not contain tumours. To mitigate this issue, Weighted Binary Cross Entropy (WBCE) was employed during training, assigning a greater weight to tumour pixels than to background pixels. In addition, Dice Score (DCS) was selected as one of the main evaluation metrics due to its robustness under class imbalance conditions (Fidon et al., 2018).

### 3.2 Workflow for small brain tumour detection in MRIs

**Figure 1:**
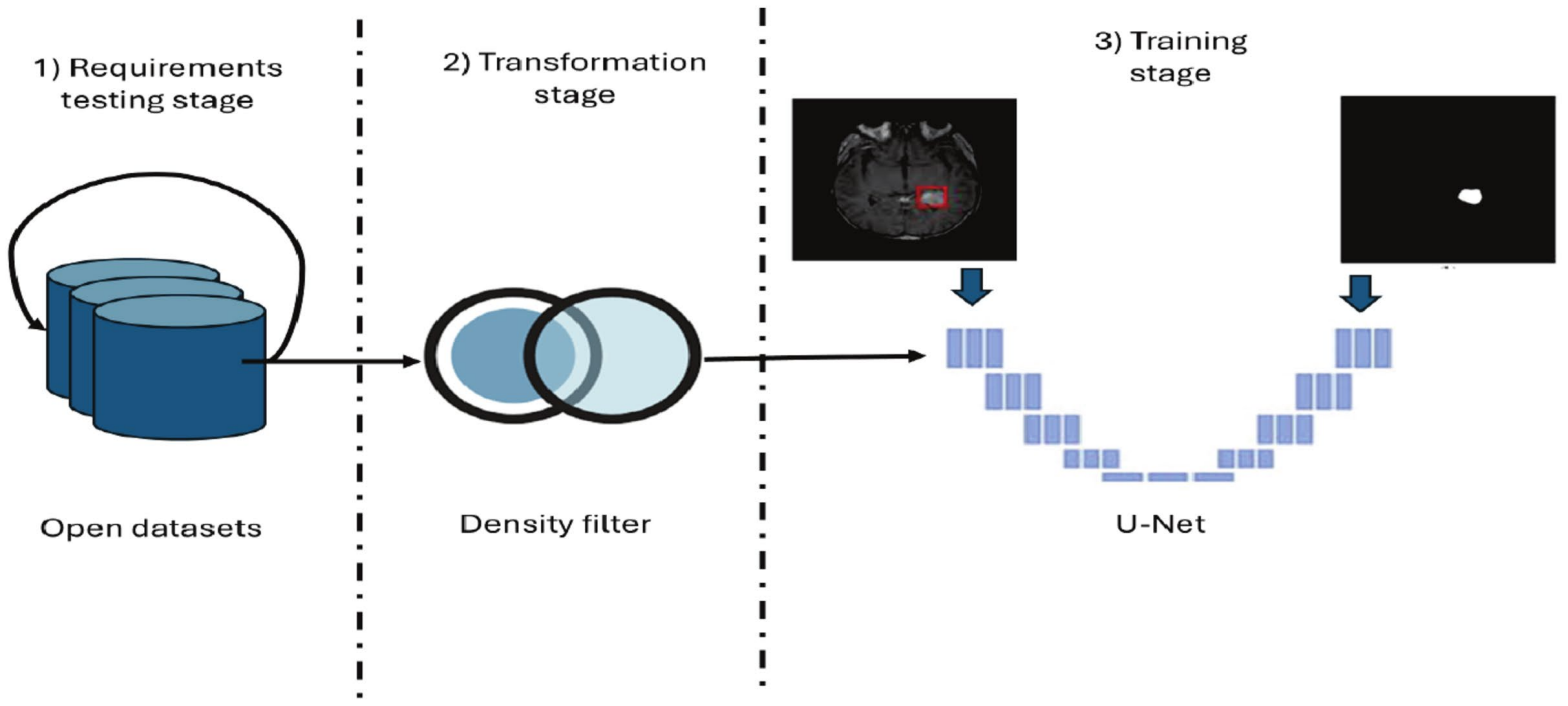
Workflow used to train small brain tumour detection models.

In this subsection, we describe the different steps applied for obtaining DL models detecting small brain tumours using MRIs. The workflow begins by ensuring that all images conform to the same standards of size and resolution. Next, a preprocessing stage applies a density filter to one of the datasets. Then, we trained two different models based on the U-Net, adding some mechanisms from the field. The model is trained with pairs for MRIs, and the image containing its mask is in black and white. This workflow is shown step by step in Figure 1 and described below. Lastly, some of the concepts used are formalised.

Normalization. It scales all image values to 0.0–1.0, reducing value influence, speeding up training, and minimising errors. Equation 1 describes it.


$$z=\frac{x-minval}{maxval-minval}$$ (1)

*Gaussian density filter*. It was introduced by Vo and Ma (2006) to adjust the probability distribution of pixel values in an image. It can increase or decrease pixel density in certain intensity ranges, thus altering the overall appearance of the image. It highlights or softens specific image areas by adjusting intensity distribution. It initially converts an image to a 32-bit floating-point data type. Then, the optical density is computed using Equation 2.


$$\text{OpticalDensity}=-\log_{10}(\frac{\text{PixelIntensity}}{255})$$ (2)

This is followed by multiplication by a specified density factor, which is set to 1 as it is the default or neutral. Subsequently, the new intensity is calculated by applying the inverse of the optical density formula. To ensure compatibility with standard image formats, the intensity is rescaled to the range of 0 to 255 by multiplying by 255. Finally, the resulting image is converted back to an unsigned 8-bit integer data type.

U-Net. Small brain tumour detection is considered an object detection task. In DL, these kinds of problems are typically solved with a U-Net model as introduced by Ronneberger et al. (2015). Karimov et al. (2019) define it as a convolutional Autoencoder, alternatively known as an “hourglass model” due to the shape. U-Net is divided into three parts: an encoder, a bottleneck, and a decoder. The encoder applies convolutional blocks, reducing the dimensionality of the input data until a small representation that includes the main features of the input. These representations are stored in the bottleneck, which is placed between the encoder and the decoder. The decoder uses deconvolutional layers to upsample the information of the bottleneck until the expected output data is obtained. In this case, the mask corresponds to the MRI in the input layer. One of the innovations of the U-Net is using skip connections, which connect opposite convolutional and deconvolutional blocks, so information that could be lost during the dimensionality reduction process could be retrieved. U-Net was chosen because it is considered the de facto model for medical image segmentation, as it is well-suited for accurate multi-scale segmentation while preserving localization precision, which is essential in our case of segmenting brain microtumors (Yao et al., 2024). Three solutions have been proposed, adding the following mechanisms to the U-Net: dilated convolutions, Spatial Pyramid Pooling (SPP), and attention mechanisms. The rationale for testing these methods is detailed below. Dilated convolutions, as stated in their seminal paper (Yu and Koltun, 2015), were explicitly developed to capture multi-scale contextual information without losing resolution, which is critical for detecting small lesions embedded in MRIs. SPP is also useful when there is a need to capture multi-scale features (Liu et al., 2023), which is relevant for tumor segmentation since tumors can vary significantly in size, shape, and location. Finally, regarding the attention mechanisms, experimental results in Oktay et al. (2018) show that they are highly beneficial for tissue and organ identification and localisation, especially for variable, small-sized organs such as the pancreas, supporting their use in our work for accurately segmenting small brain tumors.

*Dilated convolutions (DC)*: They were introduced by Yu and Koltun (2015) as applying a kernel over an area larger than its usual size by skipping pixels at a dilated rate. It is similar to convolving with a larger filter obtained by expanding the original filter with zeros, but it offers notable efficiency improvements. By allowing the network to operate on a coarser scale compared to a standard convolution, dilated convolutions are effective in capturing broader context information. Dilated convolutions are defined in Equation 3.


$$\left(F_{*l}k)(p)=\sum_{s+\mathrm{lt}}F(s)k(l\right)$$ (3)

In Equation 3, the filter is denoted by k and ∗l corresponds to the convolutional operator as l is the dilation rate. Then F(s) is a discrete function for the input data and k(l) is the application of the filter.

*Spatial Pyramid Pooling (SPP)*: It was first described by He et al. (2014) and is defined as a method that incorporates spatial structure information into pooled representations. It involves dividing the feature map into grids of cells and performing max or average pooling independently within each cell. Subsequently, these cell-based pooled representations are concatenated together to form the image representation. SPP proves highly effective for handling rigid structures, but it may not be suitable for images featuring objects exhibiting varying poses.

*Attention mechanism (AM)*: Vaswani et al. (2017) was the first paper to use attention mechanisms, which effectively grant a model the capability to “attend to” specific segments of the input sequence, prioritising those deemed more crucial or significant (Basiri et al., 2021).

Finally, we formalised the metrics used to measure the performance of the model. In this case, we are using a loss metric as WBCE and a segmentation metric as DCS. WBCE is defined in Equation 4 (Ho and Wookey, 2020).


$$J_{\text{wbce}}=-\frac{1}{M}\sum_{m=1}^{M}[\begin{array}{l} w_{x}y_{m}\times \log(h_{\theta }(x_{m}))+\\ \left(1-y_{m})\times \log(1-h_{\theta }(x_{m})\right) \end{array}]$$ (4)

If we look in-depth at the equation above, the following terms can be found: M number of training examples, w weight, $y_{m}$ target label for training example m , $x_{m}$ input for training example m , $h_{\theta }\;$ *a* model with neural network weights θ .

It is commonly used in binary classification tasks where the classes are imbalanced, meaning that one class may have significantly more samples than the other. *The purpose is to* assign different weights to positive and negative class examples during training. In our case, as regions belonging to small tumours are smaller, they are multiplied by 8. For the background of the MRIs, this multiplication is done by 1.

DCS is based on the Intersection over Union (IoU) concept, which calculates the proportion of the overlapping area between the predicted region (output image of the model) and the ground truth (image used in the output layer during training) region, relative to the total area covered by both regions (Rahman and Wang, 2016). Its values range from 0 to 1, where 0 indicates no overlap, and 1 signifies perfect spatial alignment between the regions. Formally, IoU is the ratio of the intersection area between the predicted image and ground truth to the union area of both regions, defined in Equation 5.


$$IoU=\frac{|Predictied\cap Ground\;Truth|}{|Predictied\cup Ground\;Truth|}$$ (5)

In the previous equation, |Predicted ∩ Ground Truth| denotes the intersection area between the predicted and ground truth regions, which are the common pixels. Then, |Predicted ∪ Ground Truth| represents the union area encompassing both the predicted and ground truth regions, which is all that is covered by both masks.

DCS, derived from IoU, measures the proportion of twice the overlapping area relative to the sum of the sizes of the predicted and ground truth regions (Chegraoui et al., 2021). Like IoU, it falls within the range of 0 to 1 and shares the same interpretation. Equation 6 provides its formulation.


$$\text{DCS}=\frac{2\times |Predictied\cap Ground\;Truth|}{|Predictied|+|Ground\;Truth|\;}$$ (6)

Above, the numerator corresponds to that of IoU. Subsequently, |Predicted| denotes the size of the predicted region, while |Ground Truth| signifies the size of the ground truth region.

The previous metrics provide valuable insights, but for a comprehensive understanding of the model’s performance, metrics considering True Negatives (TNs), True Positives (TPs), False Positives (FPs), and False Negatives (FNs) are essential. Specificity evaluates the ratio of TNs to the aggregation of TNs and FPs, while Sensitivity computes a similar ratio, changing TNs with TPs and FPs with FNs. Precision is determined by dividing the number of TPs by the sum of TPs and FPs. These metrics are formalized in Equations 7–9:


$$\text{Specificity}=\frac{\mathrm{TN}}{\mathrm{TN}+\mathrm{FP}}$$ (7)

$$\text{Sensitivity}=\frac{\mathrm{TP}}{\mathrm{TP}+\mathrm{FN}}$$ (8)

$$\text{Precision}=\frac{\mathrm{TP}}{\mathrm{TP}+\mathrm{FP}}$$ (9)

The interpretation of the metrics above can be done as follows. Specificity is used so the model can distinguish healthy parts of the brain from those with cancer that have not been considered without it. This metric could misclassify cancerous tissue as healthy, leading to either no treatment being recommended or treatment being applied to the wrong area. In terms of sensitivity, the model can identify most cancerous regions, though some healthy areas may be incorrectly classified as cancerous. This has consequences in spending money on erroneous treatment, which also has problems in the time dedicated to real patients. It could also happen that the physician is treating a healthy area as cancerous, which has consequences for health. The precision ensures the identification of areas of cancer among areas with tumours considered healthy. This metric is related to the fact that areas with cancer cannot be treated.

## 4 Results

### 4.1 Requirements testing stage

At this stage, it is checked that all the instances in the dataset meet some requirements that help in the standardisation of training the models. First, it has been checked that all images between datasets have the same size. In Brain T1-weighted CE-MRI and TCGA-LGG, it is 256 × 256, and for BraTS, it is 240 × 240. Apart from that, the TCGA-LGG images have been transformed into greyscale, as it has been considered that better results can be achieved.

### 4.2 Transformation stage

During this step, we used different strategies depending on the dataset to create the training sets and mitigate heterogeneity between datasets. First, two typical transformation methods have been applied: normalisation and the Gaussian density filter. The former, which is a way to reduce variations between images from different MRI scanners (Salome et al., 2023), has been applied to all the datasets. The latter showed improvements when applied to the Brain T1-weighted CE-MRI dataset (Rosa et al., 2024).

Another strategy that has been applied at this stage is the removal of upper and lower cuts of the different 3D MRIs in BraTS. This decision has been taken because this dataset only includes gliomas, so these cuts will have no tumour data and will only introduce noise.

### 4.3 Training stage

In the last step, it has been necessary to make the following decisions. First of all, the subsets for training, validation, and test have to be created by splitting the original data. The percentages to split the data have always been the same. For training and testing, percentages of 80 and 20%, respectively, have been applied. For training and validation, this split consists of 70–30%. Then, to maintain a good distribution of instances between subsets, two different strategies have been applied. For the Brain T1-weighted CE-MRI and TCGA-LGG datasets, a shuffle between instances has been applied since they are 2D MRIs; there is only one image per patient, and they are sufficiently different from each other. For Brain T1-weighted CE-MRI, the shuffle has been considered for each complete medical test. This is because they are organized in depth, so from one cut to the next, the difference can be very small and can result in data leakage. As a way to check that the models have been trained accurately, we have applied a *k*-fold validation strategy using 5 folds. We then trained the models with the different datasets. To find the optimal combination of hyperparameters, a grid search strategy with the Optuna library has been applied (Akiba et al., 2019). Grid search is a method that combines different values for the different hyperparameters of the model and finds the combination that performs the best (Bergstra and Bengio, 2012). In Table 2, all the hyperparameters and different values can be seen. The number of kernels is only specified for the first convolutional block; then, the values are doubled during the encoding stage and halved in the decoding stage.

**Table 2:** Grid search values.

Hyperparameters	Values
Number of kernels	8, 16, 32
Kernel size	2 × 2, 3 × 3
Dilation rate	2, 3, 4
Batch size	4, 8, 16
Learning rate	1 × 10^−3^, 1 × 10^−4^, 1 × 10^−5^
Number of kernels att. block	40, 50, 60
Dropout	0.3, 0.5, 0.6

### 4.4 Metrics performance with the different datasets

As mentioned before, this work aims to detect small brain tumours in MRIs using DL models. Due to the difficulty in collecting this type of data, the 3 open datasets described before have been used. The criterion applied to consider tumours as small is that of having a diameter smaller than 1 centimetre. Although to obtain the best model, it has been necessary to apply our workflow to the different datasets, the best model performs almost the same for the 3 of them. The only difference between models is the input layer when using the BraTS dataset, as it contains 3D MRIs. As part of the training process, we applied different DL mechanisms, which have led us to two main models. The former applies dilated convolutions with SPP. The latter includes dilated convolutions and attention mechanisms. Tables 3, 4 show the metrics for these models when performing at their best. The first table shows the WBCE metric in training, validation, and test. The second table compiles the same information for the DSC. For all the values, the average value of applying *k*-fold validation and the standard deviation are shown. Best results for each dataset are bolded.

**Table 3:** WBCE performance of the two proposed solutions.

Model	Training	Validation	Test
U-Net + DC + AM + BraTS	**10.0% ± 0.5**	**11.1% ± 0.5**	**14%**
U-Net + DC + SPP + BraTS	14.3% ± 1.0	14.5% ± 0.4	13.9%
U-Net + DC + AM + CE-MRI	**1.5% ± 0.3**	**1.8% ± 0.2**	**1.7%**
U-Net + DC + SPP + CE-MRI	4.4% ± 1.0	4.5% ± 0.4	4.9%
U-Net + DC + AM + TCGA-LGG	**11.1% ± 0.3**	**10.3% ± 0.2**	**10.2%**
U-Net + DC + SPP + TCGA-LGG	13.1% ± 0.9	13.6% ± 0.7	14.2%

Bolded values are best model for each dataset.

**Table 4:** DCS performance of the two proposed solutions.

Model	Training	Validation	Test
U-Net + DC + AM + BraTS	**96.3% ± 0.8%**	**92% ± 1.0%**	**91.6%**
U-Net + DC + SPP + BraTS	91.5% ± 1.7%	89.2% ± 1.5%	87.4%
U-Net + DC + AM + CE-MRI	**98.1% ± 1.2%**	**94% ± 1.0%**	**94.4%**
U-Net + DC + SPP + CE-MRI	93.5% ± 1.8%	88.5% ± 1.3%	88.8%
U-Net + DC + AM + TCGA-LGG	**90.8% ± 0.8%**	**87.3% ± 0.4%**	**86.6%**
U-Net + DC + SPP + TCGA-LGG	85.5% ± 1.9%	80.2% ± 1.7%	80.3%

Bolded values are best model for each dataset.

At this point, we obtained a model that achieves a good bias-variance trade-off. This lead as to consider the U-Net with DC and AM as the best solution. Regarding computational complexity, the proposed solution has approximately 2.40 million trainable parameters and requires about 19.8 GFLOPs per 256 × 256 forward pass, with a stored model size of roughly 29 MB. Inference takes about 35 ms per image and a training step (batch size 8) about 0.64 s on a single modern GPU, and the full 340-epoch, 5-fold schedule completes in a few hours on a single GPU, which makes the model practical as a screening aid.

Next, we analyse the best model’s performance in depth. Therefore, metrics that consider FN (a pixel that is part of a tumour and is not considered as such) and FP (a pixel not belonging to a tumour and considered as the tumour) have been used. These metrics are sensitivity, specificity, and precision, and have been compiled in Tables 5–7 for the model using U-Net+DC+AM for the training, validation, and test.

**Table 5:** Performance of the best solution with other metrics using BraTS.

Metric	Training	Validation	Test
Specificity	99.9% ± 0.1%	99.9% ± 0.1%	99.8%
Sensitivity	90.8% ± 0.5%	85.3% ± 0.5%	83.7%
Precision	83.9% ± 0.6%	84.1% ± 0.8%	82.7%

**Table 6:** Performance of the best solution with other metrics using CE-MRI.

Metric	Training	Validation	Test
Specificity	99.9% ± 0.2%	99.7% ± 0.3%	99.8%
Sensitivity	85.7% ± 0.4%	78.4% ± 0.7%	76.6%
Precision	83.0% ± 0.3%	74.2% ± 0.6%	72.2%

**Table 7:** Performance of the best solution with other metrics using TCGA-LGG.

Metric	Training	Validation	Test
Specificity	99.8% ± 0.3%	99.7% ± 0.3%	99.7%
Sensitivity	55.7% ± 1.0%	54.9% ± 0.8%	54.7%
Precision	58.0% ± 0.8%	57.2% ± 0.7%	56.7%

The results above show that at least two of the datasets perform well with the proposed solution. Thus, the next step is to compare the best model with some baselines. After reviewing the state of the art, we realised that most of the works were developed to detect brain tumours, in general, no matter the size. Therefore, the first comparison was done by retraining the models found in the state of the art, using the 3 datasets compiled in this work, and considering only tumours smaller than 1 cm, which are those that we consider of small size. The results of this first baseline comparison are compiled in Table 8. For each dataset, our best model performance against the retrained models is shown. Again, for each dataset we provide WBCE and DCS. As these works have not considered brain tumours as small, we will name these comparisons as general baselines. In these studies, Noori et al. (2019) use a U-Net with two old versions of the BraTS dataset (Díaz-Pernas et al., 2021) apply the CE-MRI trained with CNNs, and (Havaei et al., 2017) train the BraTS version from 2013 with two CNNs.

**Table 8:** Comparison of WBCE and DCS against general baselines using different datasets.

Dataset	Metric	Model	Training	Validation	Test
BraTS	WBCE	U-Net + DC + AM	**10.0% ± 0.5**	**11.1% ± 0.5**	**14.0%**
		Noori et al. (2019)	55.7% ± 0.6	56.9% ± 0.3	56.0%
		Díaz-Pernas et al. (2021)	15.3% ± 0.9	16.2% ± 0.8	16.3%
		Havaei et al. (2017)	24.3% ± 0.8	24.7% ± 0.7	25.8%
	DCS	U-Net + DC + AM	**96.3% ± 0.8**	**92% ± 1.0**	**91.6%**
		Noori et al. (2019)	9.8% ± 0.2	8.8% ± 0.1	8.9%
		Díaz-Pernas et al. (2021)	71.4% ± 0.7	71.2% ± 0.5	70.3%
		Havaei et al. (2017)	13.8% ± 0.4	12.6% ± 0.3	12.7%
CE-MRI	WBCE	U-Net + DC + AM	**1.5% ± 0.3**	**1.8% ± 0.2**	**1.7%**
		Noori et al. (2019)	41.8% ± 1.1	41.9% ± 1.5	42.4%
		Díaz-Pernas et al. (2021)	2.5% ± 0.6	2.7% ± 0.4	2.7%
		Havaei et al. (2017)	23.5% ± 0.9	22.7% ± 0.8	23.8%
	DCS	U-Net + DC + AM	**98.1% ± 1.2**	**94% ± 1.0**	**94.4%**
		Noori et al. (2019)	10.5% ± 0.3	9.3% ± 0.1	9.2%
		Díaz-Pernas et al. (2021)	81.4% ± 1.0	80% ± 0.7	80.2%
		Havaei et al. (2017)	12.1% ± 0.8	11.7% ± 0.7	11.1%
TCGA-LGG	WBCE	U-Net + DC + AM	**11.1% ± 0.3**	**10.3% ± 0.2**	**10.2%**
		Noori et al. (2019)	57.8% ± 0.5	58.9% ± 0.5	58.7%
		Díaz-Pernas et al. (2021)	19.4% ± 0.6	19.5% ± 0.4	19.9%
		Havaei et al. (2017)	30.3% ± 0.4	30.7% ± 0.3	31.8%
	DCS	U-Net + DC + AM	**90.8% ± 0.8**	**87.3% ± 0.4**	**86.6%**
		Noori et al. (2019)	8.7% ± 0.5	8.5% ± 0.3	8.4%
		Díaz-Pernas et al. (2021)	68.2% ± 0.6	67.1% ± 0.5	66.8%
		Havaei et al. (2017)	12.7% ± 0.8	11.4% ± 0.6	11.7%

Bolded values are best solution for each dataset.

Although all the tables above are for brain tumour detection, none of them is specifically focused on small brain tumours. As far as we know, only one work was developed for this purpose (Ngo et al., 2020). In our experiments, all baseline models were retrained under the same conditions and using the same datasets, but considering only tumours smaller than 1 cm according to the clinical criterion adopted in this work. Under these conditions, tumour regions occupy a substantially smaller proportion of the MRI, increasing the class imbalance problem and making segmentation significantly more challenging for the baseline models, us they were not designed for this specific task.

Therefore, in the next comparisons of the baseline, the solution proposed here is evaluated against this model. It should be highlighted that the paper only provides one metric that corresponds to the DCS obtained during the test stage. To do a more accurate comparison, a baseline has been made against this work.

**Figure 2:**
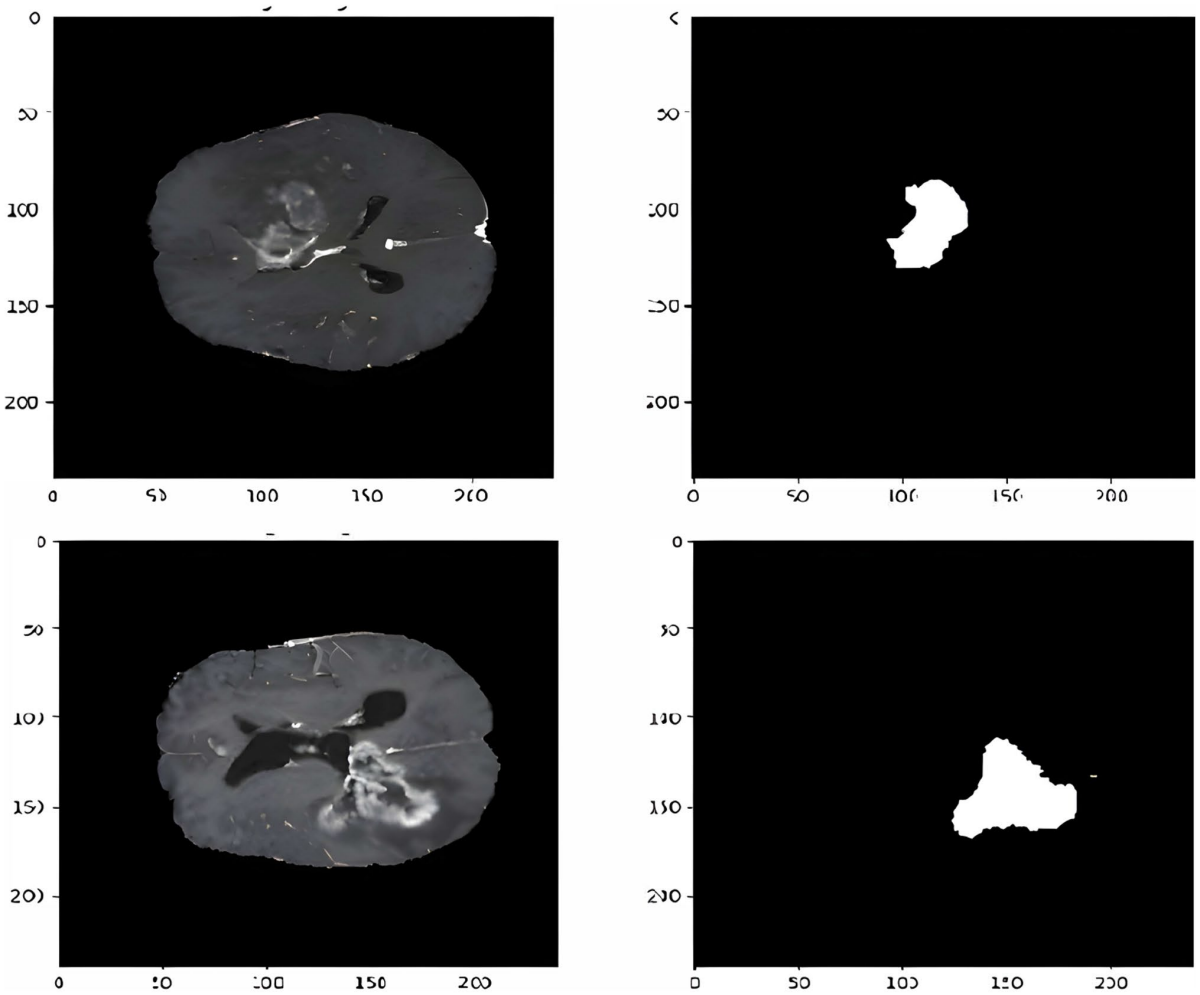
Examples of non-small tumours that have been considered as small (Amin et al., 2022).

In the authors’ opinion, there is a weakness in Ngo et al. (2020) work regarding the concept they use to define a brain tumour as small, as they state that a small tumour is 10% smaller than the median of all tumours. This criterion may introduce clinically inconsistent cases because most of the tumours in the sample are large, causing a bias and leading to a tumour being classified as small when it is not, contrary to medical judgement. In Figure 2, two examples of big tumours that were considered small by the paper are shown. The MRI is depicted on the left side, and the mask is on the right.

Thus, to carry out a fair comparison, the following experiments have been performed. The baseline model has been trained with all the datasets. For each of them, the two concepts for small brain tumours have been applied: smaller than 1 cm and smaller than 10% of the median. Table 9 depicts this baseline using BraTS to train a U-Net, as it was the dataset used in the paper, versus the proposed solution, showing the WBCE and DCS metrics.

**Table 9:** Comparison with WBCE and DCS against the baseline of small tumours using BraTS.

Metric	Model	Training	Validation	Test
WBCE	U-Net + DC + AM+1 cm diameter	10.0% ± 0.5	11.1% ± 0.5	14.0%
	U-Net + DC+AM + 10% median	**9.1% ± 0.7**	**10.1% ± 1.2**	**10.0%**
	[9] + 1 cm diameter	58.7% ± 0.3	64.4% ± 1.2	71.8%
	[9] + 10% median	45.2% ± 0.7	47.3% ± 1.2	52.4%
DCS	U-Net + DC + AM + 1 cm diameter	96.3% ± 0.8	92.0% ± 1.0	91.6%
	U-Net + DC + AM + 10% median	**98.9% ± 0.6**	**96.1% ± 0.3**	**96.8%**
	[9] + 1 cm diameter	13.1% ± 0.5	10.5% ± 0.2	9.6%
	[9] + 10% median	35.4% ± 0.4	31.3% ± 0.1	32.0%

Bolded values are best model plust clinical criteria for each metric.

As can be observed, the solutions detailed in this paper perform better. Then, depending on the filter for small brain tumours, there are some slight differences. Finally, for a full comparison, it is necessary to measure how the proposed solution performs against the baseline of small brain tumours trained with the rest of the datasets and applying the medical criteria for small tumours (<1 cm). Table 10 shows these results for both metrics.

**Table 10:** Comparison with WBCE and DCS against small tumours baseline CE-MRI, and TCGA-LGG.

Dataset	Metric	Model	Training	Validation	Test
CE-MRI	WBCE	U-Net + DC + AM + 1 cm diameter	**1.50% ± 0.3**	**1.80% ± 0.2**	**1.7%**
		[9] + 1 cm diameter	69.3% ± 0.4	70.2% ± 0.6	70.1%
	DCS	U-Net + DC + AM + 1 cm diameter	**98.1% ± 1.2**	**94.0% ± 1.0**	**94.4%**
		[9] + 1 cm diameter	25.5% ± 0.5	21.0% ± 1.0	20.3%
TCGA-LGG	WBCE	U-Net + DC + AM + 1 cm diameter	**11.1% ± 0.3**	**10.3% ± 0.2**	**10.2%**
		[9] + 1 cm diameter	68.2% ± 0.5	68.5% ± 0.7	69.2%
	DCS	U-Net + DC + AM + 1 cm diameter	**90.8% ± 0.8**	**87.3% ± 0.4**	**86.6%**
		[9] + 1 cm diameter	27.9% ± 0.2	22.5% ± 0.5	23.1%

Bolded values are best model plust clinical criteria for each metric and dataset.

Finally, as a way to quantify the contribution of the architectural components, a *post-hoc* ablation study was conducted on CE-MRI, the most stable and best-performing of the three datasets. Three architectural variants were evaluated progressively. First, a plain U-Net without any additional mechanism obtained a WBCE of 12.9% ± 14.2, 40.3% ± 5.6, and 39.9%, and a DCS of 87.1% ± 14.2, 59.7% ± 5.6, and 60.1% in training, validation, and test, respectively, showing a large gap between training and validation that indicates overfitting and poor generalisation. Second, the addition of DCs and SPP (U-Net + DC + SPP) substantially reduced this gap, achieving a test DCS of 88.8% and a test WBCE of 4.9% with good generalization, as reported in Tables 3, 4. Third, replacing SPP with the AM (U-Net + DC + AM) further improved performance to a test DCS of 94.4% and a test WBCE of 1.7%, representing the best overall result. These results confirm that DC is the primary driver of generalisation, reducing the train-test DCS gap from approximately 27 points in the plain U-Net to under 4 points, while the AM provides the largest incremental gain in absolute segmentation performance.

### 4.5 Detailed description of the best solution

**Figure 3:**
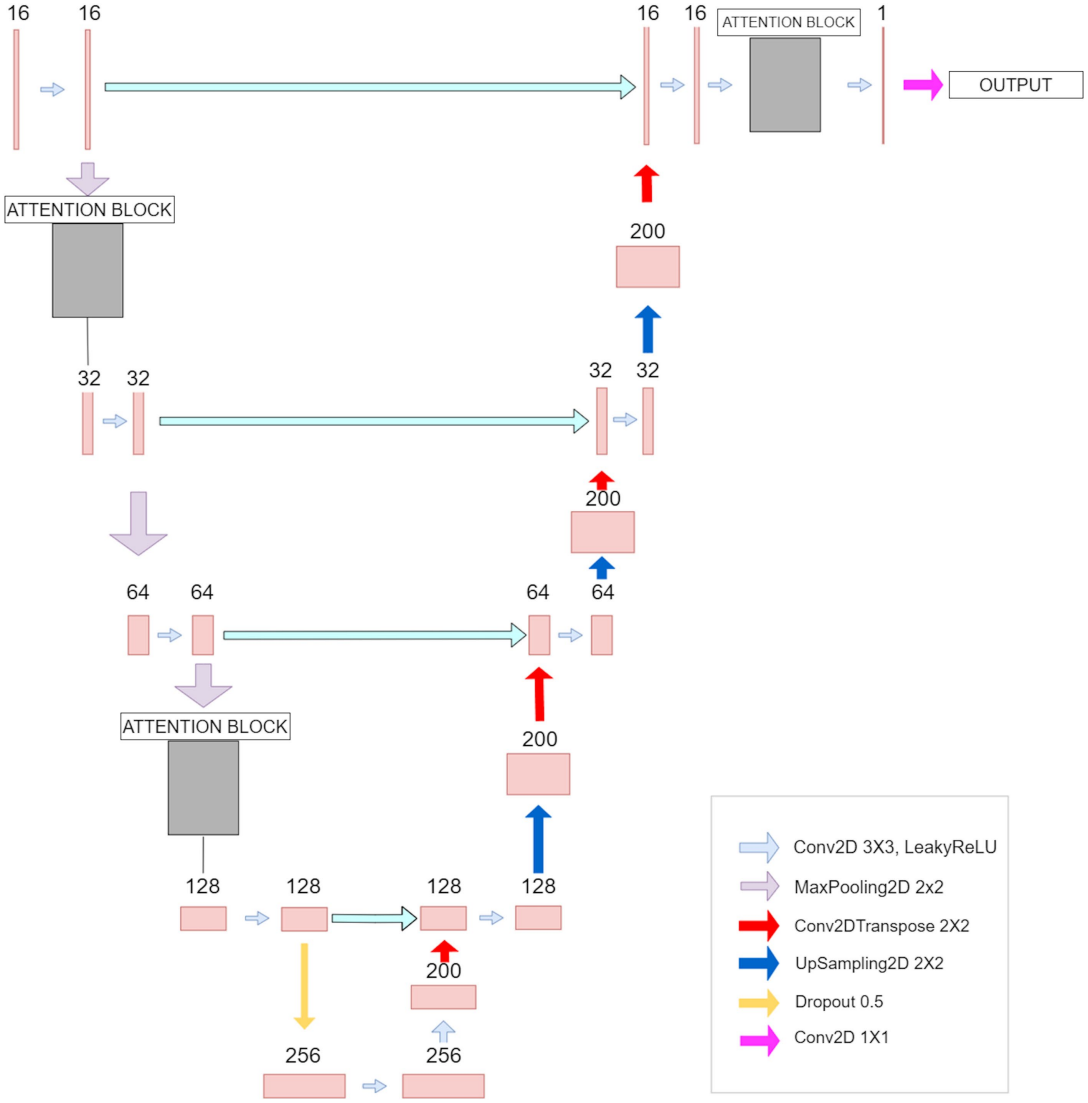
Architecture for small brain tumour detection with MRIs.

After the different comparisons, it is observed that one of the proposed solutions always performs better than the baselines, regardless of the dataset or the criterion used to consider a brain tumour small. This solution is a U-Net using DCs and AMs, as depicted in Figure 3.

The model has 4 convolution blocks in the encoder and 4 in the decoder. In the encoder, each block is composed of two convolutional layers, followed by a maxpooling (typically used in greyscale images). It should be noted that from one block to the next, the number of kernels per layer is doubled. In addition, after the first and third convolution blocks, there is an attention module. The output of the fourth block is used as input to the bottleneck of the U-Net.

As for the decoder, each convolution block has a transposed convolutional layer, an upsampling layer, a concatenation layer, and, at the end of the last block of the decoder, an attention module. Then its attention mechanism is connected to a last 1 × 1 convolution, which will be the model output. All the activation functions are LeakyReLU, except in the last layer, which is sigmoid. This allows the model to classify the two types of pixels: those belonging to the mask and those not.

The size of the kernels is 3 × 3, keeping the existing padding, and the number of neurons follows this sequence: 16-32-64-128 in the encoder, 256 for the bottleneck, and 128-64-32-16 in the decoder.

Finally, there are skip connections that go from the blocks of the encoder to their opposite block in the decoder, allowing the recovery of information that has been lost during the process of encoding.

### 4.6 Experts’ evaluation of the model

To analyze the proposed model in depth, an evaluation was carried out by a medical expert specialized in brain tumour diagnosis and treatment. The expert was a radiation oncologist (co-author of this work) at the Radiation Oncology Department of Ramón y Cajal Hospital (IRYCIS), with more than 8 years of clinical experience in the evaluation, delineation, and treatment planning of primary and metastatic brain tumours using magnetic resonance imaging.

**Figure 4:**
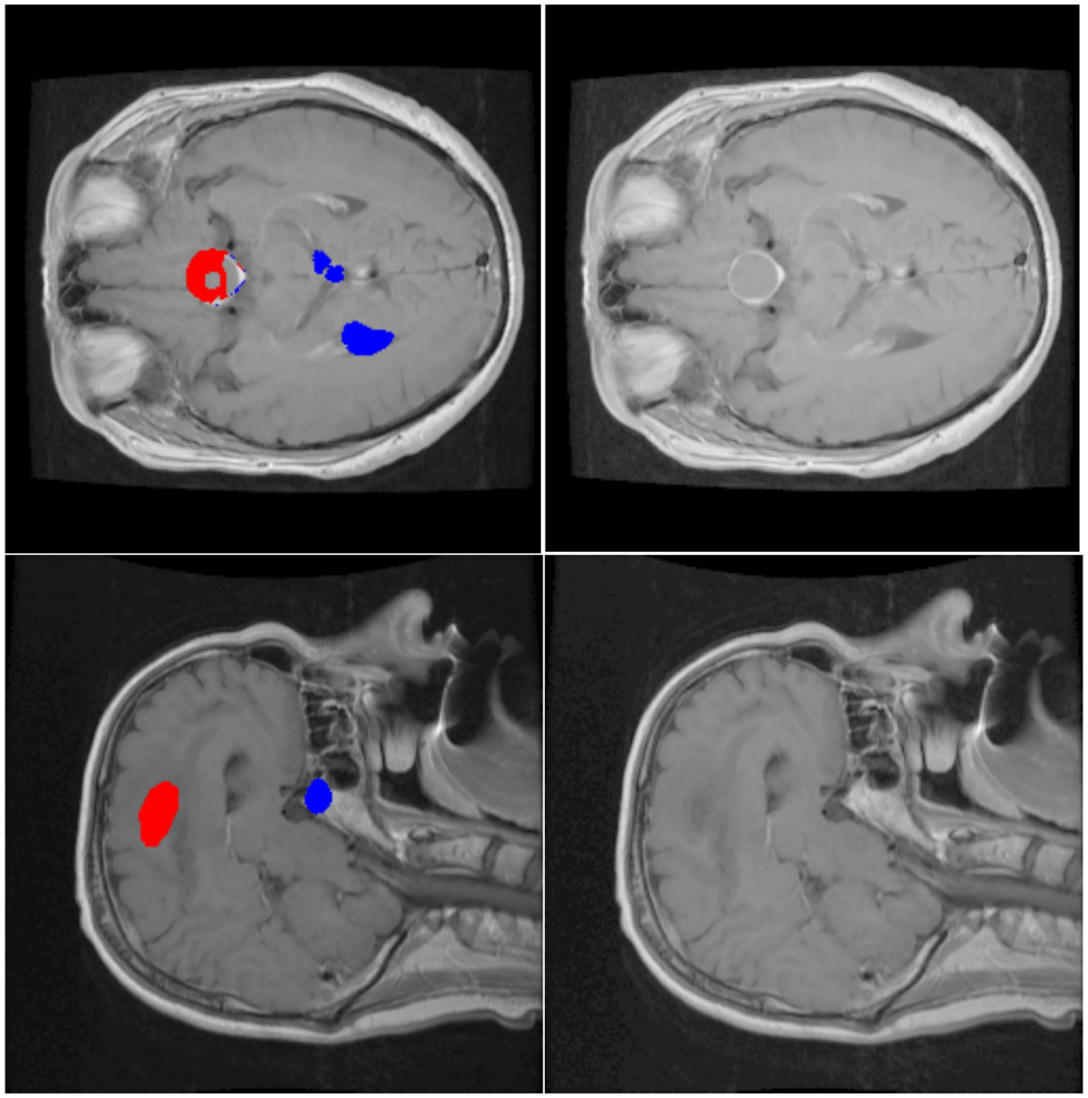
Examples of MRI with FPs and FNs.

This evaluation has two parts: one that assesses the differences in model performance across the datasets used and a more exhaustive one that analyses each of the instances of the test set for the best of our approaches, which is CE-MRI, as the most stable in all experiments and metrics performed. For this dataset, the test set was split into quartiles within the same range, based on the best and worst DCS values. Then, each instance was evaluated following a sequential blind protocol: the expert first reviewed the original MRI independently, without access to the model output, and subsequently compared the findings with the predicted mask and the ground truth mask to assess FP and FN. These classifications were based on the expert’s visual clinical judgment, consistent with standard radiological practice, rather than on a predefined quantitative threshold. The evaluation was conducted across multiple sessions, with cases generating diagnostic uncertainty reviewed in additional sessions until a definitive assessment was reached. No formal intra-rater consistency check was performed. Four cases from the test set were flagged as unevaluable and excluded from the quantitative agreement analysis. In one case, the model detected a cerebral gyrus (normal brain anatomy) rather than a lesion. In the third case, the model confused the choroid plexus (a normal anatomical structure) with a tumour. In two cases, no lesion was identifiable. These four cases were retained in the qualitative discussion as informative failure examples illustrating the types of anatomical ambiguity that challenge the model. Figure 4 shows a pair of examples of FPs (blue pixels) and FNs (red pixels).

**Figure 5:**
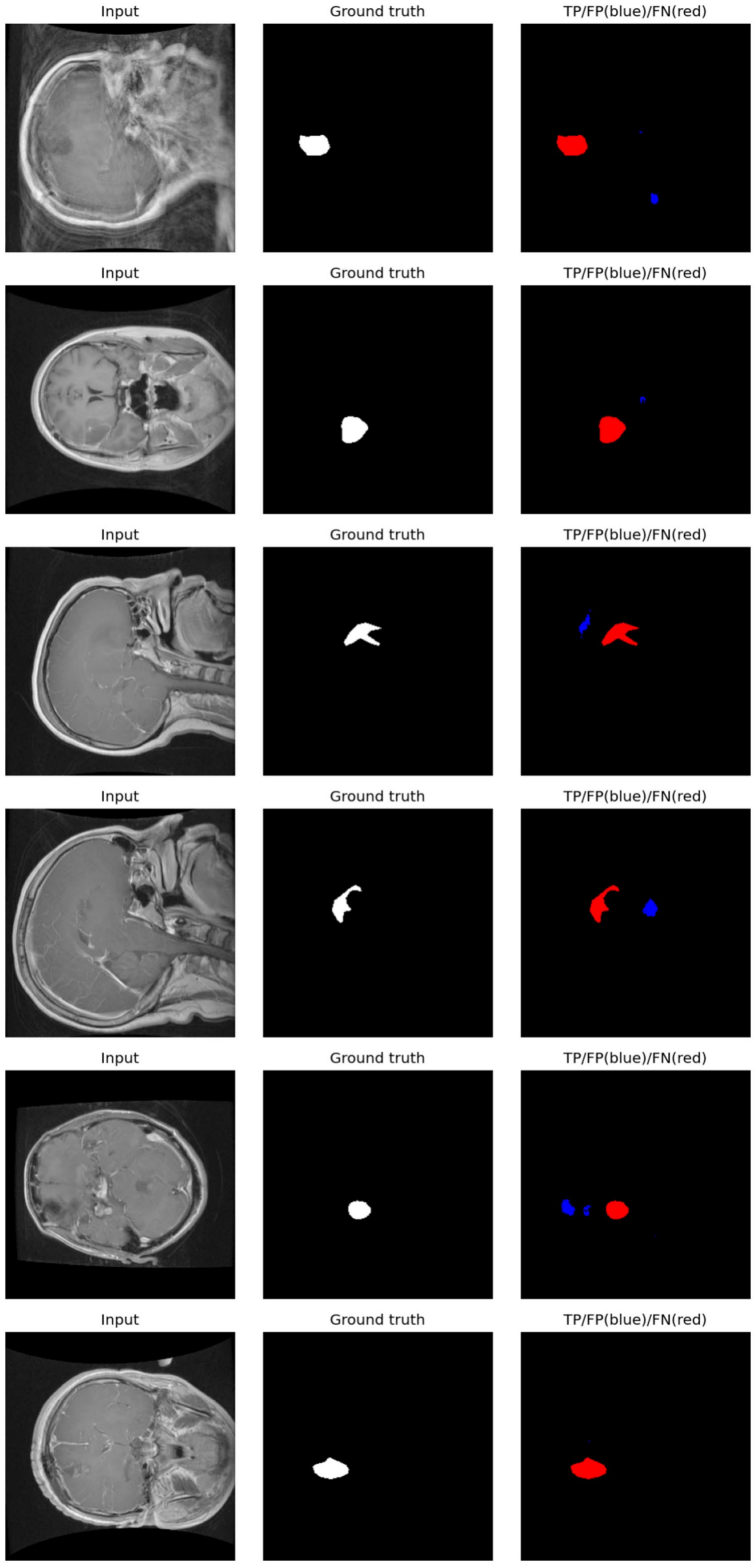
Lowest-dice test cases.

In contrast with the previous figure (Figure 4), Figure 5 presents the six lowest-DCS cases from the CE-MRI test set, grouped by the dominant error type. Three recurring failure modes emerge: (i) FN dominance (rows 1, 2, 6) in small, round lesions with weak gadolinium enhancement, where the model under-segments or misses the tumour core entirely; (ii) boundary fragmentation (row 3) in tumours with irregular, branching morphology; (iii) FP leakage (rows 4, 5) in crescent-shaped or heterogeneous lesions adjacent to high-intensity anatomical structures, where the model over-extends the segmentation into surrounding tissue.

The first part of the evaluation concluded that the datasets are highly heterogeneous, comprising various image sequences. Additionally, they include different types of primary, benign, and malignant tumours, such as pituitary adenomas, meningiomas, and gliomas. This heterogeneity poses challenges in developing a tumour-specific model; however, it allows for broader applicability across tumour types. Other factors that may influence the results include the type of contrast agent used, acquisition protocols, equipment characteristics, slice thickness, and the reconstruction techniques applied.

**Figure 6:**
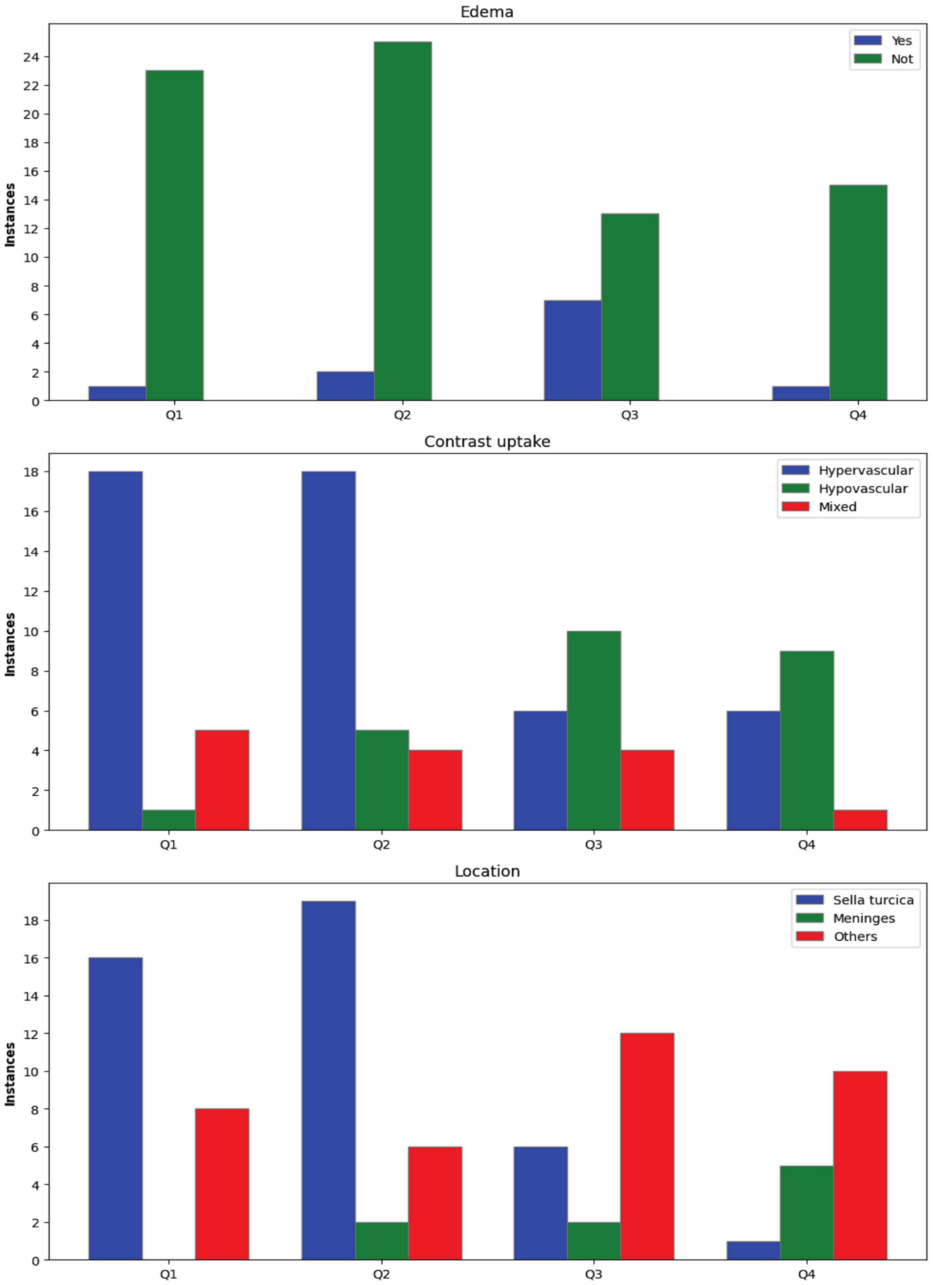
Experts’ evaluation of the best approach.

During the second part of the evaluation, three important features have been considered: the quantity of the oedema, the type of lesion according to its contrast uptake, and the location. For oedema, it is fundamental in the diagnosis of brain tumours because it influences the treatment decision and the monitoring of tumour response and evolution. Also, correct assessment of oedema allows for better interpretation of images and more informed decision-making in patient management. Differentiation of brain tumours according to their sequence and contrast uptake is essential for a complete evaluation of the tumour, since it allows safer surgical planning, influences the selection and effectiveness of treatment, and provides vital information about the patient’s prognosis. Finally, accurate identification of the location of the brain tumour is essential for accurate diagnosis, treatment planning, prognostic assessment, and monitoring of the patient’s progress. It helps to correlate symptoms with affected areas, choose the appropriate treatment, and anticipate possible complications or sequelae. All this information has been compiled in Figure 6, which shows a bar diagram for each of the three evaluated features across the four DCS-based quartiles. For edema, values are coded as present or absent. For contrast uptake, values are coded as hypervascular, hypovascular, or mixed. For location, values are coded as sella turcica, meninges, or other. Annotations were performed by the expert radiation oncologist co-author following a predefined clinical protocol applied to all 264 instances of the CE-MRI test set.

To quantify the agreement between the model and the expert, a detection-level analysis was performed on the CE-MRI test set (264 cases). Each case was classified as lesion-positive or lesion-negative by both the model and the expert, yielding 236 TPs, 13 FPs, 11 FNs, and 4 TNs, with an overall observed agreement of 90.9%. This indicates strong consistency between the model and the clinical expert.

Then, a quantitative assessment of FPs and FNs for the CE-MRI test set (13 FP and 11 FN cases out of 264 total test instances) was included in Table 11. Among FP cases, 9 (60%) were considered clinically understandable due to anatomical ambiguity, including 1 case in which a cerebral gyrus was mistaken for a lesion and 1 case in which the choroid plexus was misidentified as a tumour. Among FN cases, 10 (67%) involved lesions with low contrast or overlap with peritumoral edema.

Information from Table 11 is complemented with Table 12, analysing CE-MRI ‘s dataset applying tumour identification, differentiating between glioma, meningioma, and pituitary adenoma using the DSC. When evaluating image sets with this metric, tumours were classified by location from highest to lowest similarity into quartiles. In the studied series, most tumours were in Q1, followed by Q2, Q4, and Q3.

**Table 11:** Analysis of false positives/negatives and their clinical impact.

Tumour feature	# (%) of FP cases	# (%) of FN cases
Location		
Sella turcica	2 (15%)	1 (10%)
Meninges	1 (7%)	3 (30%)
Other locations	10 (78%)	6 (60%)
Contrast uptake		
Hypervascular	4 (30%)	2 (20%)
Hypovascular	5 (38%)	7 (70%)
Mixed	4 (32%)	1 (10%)
Oedema		
Present	8 (62%)	9 (80%)
Absent	5 (38%)	2 (20%)
Total	13 (100%)	11 (100%)

**Table 12:** Analysis of DCS quartiles.

Quartile	Instances per quartile	# (%)
Q1 (85–100% DCS)	128/264	48.5%
Q2 (70–85% DCS)	75/264	28.4%
Q3 (55–70% DCS)	20/264	7.6%
Q4 (<55% DCS)	41/264	15.5%
Total	264	100%

## 5 Discussion

Some of the results described above are described here. First of all, the proposed solutions are discussed after being trained with the different datasets from the state of the art. As can be seen in Tables 3, 4, the best solution is always the U-Net using the DCs and AMs. Regarding the dataset that obtains the best results, this is the one called CE-MRI, which makes sense as this is the one with the most medical tests. In terms of the segmentation metrics used to train the models, it seems that both overfitting and underfitting are avoided. The best solution, the model accomplishes the variance-bias trade-off (Belkin et al., 2019). For the bias, it should surpass the performance of a human doing the same task, which varies a lot depending on the case, going from 78 to 80% (Jordan et al., 2003). The variance depends on the differences between the metrics of the training, validation, and test stages, which, in the worst case, is around 4. Looking at the standard deviations, they are all very low, indicating that the solutions are very stable. Therefore, it can be concluded that the proposed solutions performed well, with the U-Net using DCs and AMs performing best.

To have an in-depth interpretation of the best model’s performance, metrics of sensitivity, specificity, and precision are obtained and compiled in Tables 5, 8, 9. By considering all the tables at the same time, it can be highlighted that specificity is consistently high, indicating that healthy tissue is correctly identified in most cases despite the severe class imbalance. For sensitivity, in BraTS and CE-MRI, there are some problems in considering parts of the tumours as healthy, not highly precise. For TCGA-LGG, this metric is around 50%, so it covers half of the tumorous area. Finally, precision is a little bit high in all the cases except TCGA-LG. Thus, the model is generally able to localise tumour regions with good precision, although difficult cases remain challenging, particularly in TCGA-LGG. These lower values compared to BraTS and CE-MRI are directly attributable to the compound class imbalance present in that dataset. At the image level, 2,556 of the 3,929 total images contain no tumour at all, meaning that after filtering for small tumours, the usable training subset is substantially smaller than for the other datasets. At the pixel level, the tumour regions in the retained images are among the smallest across all datasets. This double imbalance, few positive images and very few positive pixels within them, limits the model’s ability to learn reliable tumour representations despite the mitigation strategies applied, and explains the performance gap relative to BraTS and CE-MRI. The evaluation of the TCGA-LGG cases leads to the conclusion that this model is useful as an initial screening tool for small lesions and that further expert review is required. A higher number of false positives than false negatives is acceptable, and it is a useful initial screening tool with subsequent revision by experts.

Class imbalance had a direct impact on model performance, particularly regarding sensitivity. While specificity remained consistently high across all datasets due to the predominance of background pixels, lower sensitivity values indicate difficulties in detecting all tumour regions, especially small and hypovascular lesions. This effect was more evident in TCGA-LGG, which contains a high number of non-tumour MRIs and heterogeneous tumour appearances. Although no data augmentation techniques were applied in this work, the proposed architecture still achieved strong segmentation performance across multiple datasets. It should be noted, however, that these mitigation strategies (WBCE together with the DCS) attenuate but do not fully eliminate the impact of class imbalance, particularly in the compound case of TCGA-LGG.

The three datasets also differ substantially in their acquisition characteristics, which contribute to the performance differences observed across them. BraTS 2021 provides multi-modal volumetric scans (T1, T2, T1-CE and FLAIR); the Brain T1-weighted CE-MRI dataset is single-sequence contrast-enhanced 2D, and TCGA-LGG is 2D T1-weighted, each acquired on different scanners and protocols and with different resolutions and slice thicknesses. These differences amount to a domain shift: intensity statistics, contrast, and lesion appearance are not identical across acquisition regimes, which, together with the dataset-specific class imbalance discussed above, helps explain why segmentation quality is highest on the more homogeneous, contrast-enhanced CE-MRI and BraTS data and lowest on the heterogeneous, lower-contrast TCGA-LGG cohort. That a single architecture, trained per dataset under identical settings, performs consistently across these regimes provides partial evidence of robustness; nevertheless, a dedicated cross-dataset and domain-adaptation analysis (training on one regime, testing on another) is required to quantify the domain-shift effect directly.

Once it has been demonstrated that a well-performing model has been obtained, it needs to be compared with the state of the art. Table 10 compares our best model with some of these architectures that have been retrained with the 3 datasets used in this paper and considers our medical criterion for small brain tumours. As can be seen, the performance of the models when considering only small brain tumours is not very good. Only the architecture presented in Díaz-Pernas et al. (2021) obtains similar results, but with differences of 25 and 1% for DCS and WBCE for the best case. The fact is that many of the compared methods rely on global contextual information and were originally evaluated using tumours of varying and often large sizes. Consequently, when restricted to very small lesions, these models tend to over-segment background regions or fail to identify tumour areas correctly, leading to very low DCS. In addition, overlap-based metrics such as DCS are highly sensitive to segmentation errors in very small lesions, where even small boundary inaccuracies can produce substantial metric reductions.

Finally, another comparison against the only paper achieving the specific task of detecting small brain tumours in MRIs has been done. The comparison using BraTS, see Table 11, shows that our architecture always performs better, but it obtains the best results using the filter of the 10% of the median for small brain tumours. Considering that this criterion considers a lot of non-small brain tumours as small and the bias of the results when including tumours of bigger areas, this difference is relatively small and points out our model as more accurate. To do a complete comparison, the performance of this model considering the criterion of small brain tumours with less than 1 centimetre has been measured. Results were shown in Table 12, and again, our model obtained better values. These huge differences with Ngo et al. (2020) are consistent, as reported DCS are 44.99 and 38.54% for tumours smaller than 2,000 and 1,000 voxels, respectively, despite using a dedicated architecture specifically designed for small tumour detection under a different small-tumour definition criterion. This paper also reports that DCS decreases substantially as tumour size becomes smaller, highlighting the intrinsic difficulty of microlesion segmentation.

To confirm that the reported comparisons are not attributable to sampling variation, we also estimated 95% bootstrap confidence intervals for the per-image DCS on the test sets; the performance differences with respect to the baselines (Tables 10–12) are far larger than these intervals. Concretely, the proposed model’s per-image DCS has a mean of 71.4% with a 95% bootstrap confidence interval of [67.8, 74.8%] on CE-MRI (*n* = 264) and 78.0 [72.7, 83.1] on TCGA-LGG (*n* = 134). Because the gaps with respect to the general-purpose baselines (e.g., 96.8% vs. 9.6% DCS) greatly exceed these intervals, those differences cannot be attributed to sampling variation.

Regarding the evaluation of experts, in the first part of it, which evaluates the differences between models trained with different datasets, very heterogeneous datasets with different image sequences are observed. In addition, different types of primary tumours, benign and malignant, are included, including pituitary adenomas, meningiomas, and gliomas. This makes it difficult to get a specific model, but on the other hand, it allows its use for all tumours. If re-trained by tumour type, it is possible that detection could be improved independently. Other factors that may be influencing this are the type of contrast, the acquisition protocol, the acquisition unit and characteristics, the slice thickness, and the reconstructions performed. It should be taken into account that clinically, the evaluation of MR images of gliomas is more complex than that of other lesions, such as meningiomas or pituitary adenomas, due to their imaging characteristics with great heterogeneity in the uptake of gliomas and differentiation between high and low-grade gliomas. This may influence the poorer evaluation in series that include a large proportion of these cases.

For the evaluation of the best approach compiled in Figure 6, it should be considered that not all the quartiles have the same number of instances. The first one has 23, the second has 27, the third has 20, and the last one has 16, considering that 4 instances have been discarded due to the very bad results provided by the model.

Examining the oedema analysis, all quartiles show similar proportions, with significantly more instances that do not present oedema. This is more significant in the first and second quartiles. It should also be highlighted that the third quartile is the one with a higher distribution between having oedema or not, being the case with more instances for the first class and the least populated for the second one. This may lead to results in Table 8, where the model experienced a lower sensitivity, which means confusion between oedemas and tumours. As stated in Thiele et al. (2024), this is a challenging problem that could arise due to the contrast of the medical image (Galldiks et al., 2024). In medical terms, this could be oedema related to tumour aggressiveness and tumour size. Lesions with greater oedema present more complications and neurological alterations than lesions with no or more limited oedema (Liyanage et al., 2020).

Figure 6 shows that, regarding the feature related to contrast uptake, the first two quartiles are very imbalanced between classes, having a more equitable distribution in the third and fourth quartiles. In the first two, a greater presence of hypervascular contrast by far, with no big differences between quartiles, is observed. In the other two quartiles, there is a greater presence of the hypovascular class, also with small differences between them. Again, these results could explain the lower sensitivity in Table 8, as it is usually easier to detect hypervascular lesions than hypovascular lesions (Khadem et al., 2012). The main reason for these problems is that hypovascular lesions have similar characteristics to the brain, which is more hypovascular. The class related to mixed contrast has the same instances in all the quartiles except in the worst one. From a medical point of view, this may be due to the presence of different tumour types, as mentioned above, the uptake of gliomas is heterogeneous with areas of hypo- and hypervascularisation, which may affect the segmentation performance of the model. Finally, we can conclude that the lack of contrast limits the ability to identify lesions and is evident in false negatives, where lesions are more difficult to define, failing to identify lesions where they exist.

In Figure 6, the evaluation related to the location of the tumours was done. Again, there are some patterns before a comparison of the first two quartiles with the last two. In the first group, there is a greater presence of tumours located in the sella turcica by far. It should also be highlighted that there is no presence of tumours in the meninges for the first quartile. In the last two quartiles, the tumours located in other areas of the brain are the ones with the most instances. This makes the model have more false positives due to the difficulty of this task, leading to the sensitivity values in Table 8. Analysing the medical consequences, the tumour location can help in the diagnosis, especially in meningeal and sella turcica tumours, which are typically located in these regions. Gliomas often occur in the cerebral hemispheres or posterior fossa in adolescents and children.

Regarding Table 6, the following results are obtained. FP: 9 (60%) were considered clinically understandable (e.g., anatomical ambiguity), 6 (40%) were flagged as potentially misleading, and among FN: 10 (67%) involved lesions with low contrast or overlap with edema, and 5 (33%) were missed despite adequate visibility, suggesting model limitations. Of all cases, 20 (67%) were judged to have limited clinical impact, while 10 (33%) were considered important for refinement. These findings support future work on targeted detection strategies (e.g., for hypovascular lesions). This information is complemented by Table 7, where differentiating between glioma, meningioma, and pituitary adenoma was achieved in 48.5% of cases with a DCS greater than 85, and 28.4% with a DCS between 70 and 85%. The results with a DCS lower than 70% were 23.1%.

Apart from that, it should also be noted that four particular use cases were found in the last quartile where the model made a diagnosis that could not be evaluated. In three of them, the model does not identify the lesion, and, in particular, in one case where a zirconvolution (regular anatomy of the brain) is detected. In the other case, the model confuses the choroid plexus with a lesion. It is relevant to know the false positive and false negative rates of the model. If the model says that there is a tumour and there is not, an unnecessary treatment for the patient could be applied, which is not free of side effects. If the model says there is no tumour and there is, it could have doubled or tripled in size, and less curative therapeutic options. For all these reasons, it is necessary to know the error rate and to be able to act accordingly.

If we connect the results from Table 8 with the experts’ evaluation, this model would be very useful to help in the diagnosis of small tumours, serving as an initial filter for doctors who would then, based on the detection of the model, carry out a detailed study of the identified area, screen the identified area, and determine the definitive diagnosis.

## 6 Limitations

The main limitation of this work relates to the implications of applying AI techniques in real-world clinical environments. In this regard, as this is an initial exploratory approach, we emphasise that this study aims to investigate how deep learning models could support the task of small brain tumour segmentation using MRI.

A limitation that does not allow the proposed solution to be fully generalised is the absence of external validation on an independent multi-institutional dataset. While the three datasets used in this work, BraTS 2021, CE-MRI, and TCGA-LGG, span heterogeneous tumour types, MRI sequences, imaging dimensionalities, and acquisition sources from different institutions and countries, none of them constitutes an independent prospective validation cohort. This heterogeneity was intentionally retained to provide a partial proxy for the variability encountered in real-world clinical settings, and the consistent performance of the proposed model across these datasets provides initial evidence of robustness. However, prospective validation on data from institutions not represented during training is required before any claims of clinical generalisability can be made. The curation of a dedicated dataset for clinically defined small brain tumours (<1 cm) and its use in a multi-centric evaluation constitute the most immediate next step in this line of work. We note that several public datasets released around the time of this study could serve as partial external validation cohorts. Examples include a multi-institutional meningioma segmentation dataset (LaBella et al., 2024), a large open-access brain metastasis dataset containing sub-centimetre enhancing lesions (Ramakrishnan et al., 2024), and the BRISC multi-source brain tumour dataset (Fateh et al., 2026). However, each dataset covers only a single tumour type, differs in imaging modality, or partially overlaps with the sources used here. Therefore, none currently provides an independent cohort that matches our inclusion criteria of lesions ≤ 1 cm across all studied tumour types.

A further limitation concerns the expert clinical evaluation protocol. Although annotations were reviewed by a specialist radiation oncologist following a structured sequential blind protocol and a predefined assessment of edema, contrast uptake, and tumour location, a formal quantitative inter-observer agreement analysis between two independent annotators was not performed for the full cohort. This reflects the retrospective nature of the study and the constraints of the clinical setting.

A final limitation concerns the use of dedicated Explainable Artificial Intelligence (XAI) techniques. Although the proposed architecture incorporates AM that improves the localisation of relevant tumour regions, and the segmentation outputs were clinically evaluated by a medical expert, *post-hoc* XAI methods such as Grad-CAM or Integrated Gradients were not applied in this work. The exploration of such techniques to further improve the interpretability and clinical transparency of the model is planned as the immediate next step.

Datasets include a wide range of tumours such as pituitary adenomas, meningiomas, and gliomas. While this heterogeneity makes it challenging to develop a tumour-specific model, it also allows the model to be applied broadly across different types. Retraining the model for each tumour type could further improve detection performance for individual cases.

It is crucial to include the MRI sequences commonly used in brain tumour diagnosis, at a minimum, T1-weighted, T2-weighted, and flow sequences, as they enable the reliable detection of small lesions by the models employed. Additionally, details about the imaging equipment, including the use of gadolinium-based contrast agents, are essential. Other influential factors include the type of contrast agent, acquisition protocol, scanner unit and its technical specifications, slice thickness, and any post-processing or reconstructions performed.

As this study supports problem-solving in clinical practice for the treatment of brain tumours, the model could be further retrained for the detection of small metastases, a relevant clinical need given the paradigm shift in managing patients with multiple metastases in recent years. Before the advent of radiosurgery, patients with multiple metastases were typically treated with whole-brain radiotherapy (Vogelbaum et al., 2022). However, following technological evolution and improved systematic treatments, the use of radiosurgery in the treatment of up to 15 brain metastases has become widespread, demonstrating adequate disease control and improved neurocognitive test results (Hughes et al., 2019). This places adequate detection of small lesions that are candidates for radiosurgery treatment in a crucial situation.

## 7 Conclusions and outlook

This study is considered a first approach to exploring how medical environments could benefit from applying Deep Learning techniques to detect small brain tumors in MRI scans. Initially, we tested various approaches to identify a solution with optimal segmentation performance. This process revealed that employing a U-Net architecture with dilated convolutions and attention mechanisms yielded the best performance. Subsequently, we evaluated this model using metrics that measure false positives and false negatives across different datasets. Notably, critical values were only detected when using the TCGA-LGG dataset. Next, we compared our solution with three baseline models designed for detecting brain tumors in general, rather than specifically small tumors. Our approach consistently outperformed these baseline methods. Furthermore, we compared our model with the only state-of-the-art approach specifically designed to detect small brain tumors. Even when adopting the criteria defined in that work, which considers tumours small if they are 10% of the median tumour size, our solution achieved better results. When applying the medical criterion for small tumours (1 cm in diameter), our model significantly outperformed the results of the work. An additional key contribution of this work is an expert evaluation conducted by a medical specialist. The evaluation highlighted challenges with false negatives, which stem from classical issues faced by clinicians, such as distinguishing between tumours and oedemas, as well as confusion caused by hypovascular areas. An ablation study on CE-MRI confirmed that DCs are the primary driver of generalisation, reducing the train-test DCS gap, while the AM provides the largest incremental gain in absolute segmentation performance.

As demonstrated, this research represents an initial step towards its eventual future application in clinical practice. We have proposed the evaluation of a redefined clinical concept and assessed its viability with existing models and available datasets. The next immediate step is that of collecting and curating a richer and more extensive dataset that better captures the variability in the input distribution of small tumors and that contains annotations that add to the explainability of edge or unclear cases. Critically, this multi-centric evaluation will serve as the external validation step needed to rigorously assess the generalisability of the proposed architecture across institutions, scanners, and acquisition protocols not represented in the current datasets. Then, we plan to move to a multi-centric evaluation that incorporates the requirements for trustworthy AI that are fundamental to transitioning research to clinical practice that complies with existing ethical and regulatory frameworks. As the final step in the clinician’s part, we plan to integrate explainability techniques to improve the model’s interpretability. This will help clinicians better understand the model’s predictions and build trust for its potential application in real-world clinical settings.

From a technical perspective, several research directions emerge from this work. First, we aim to develop models better suited for early screening scenarios, particularly by improving differentiation between edema and tumour tissue, and between hypovascular and hypervascular lesions. We also plan to expand detection beyond common tumour locations (e.g., sella turcica, meninges) to include rarer and more anatomically variable tumour types, enhancing the model’s generalizability across real-world clinical cases. Finally, future work will explore dedicated XAI methods to further improve interpretability and clinical transparency.

One promising path involves a two-stage architecture, where the first module performs sensitive region detection, flagging potentially abnormal areas, and the second performs fine-grained classification to distinguish tumour subtypes or rule out benign anomalies. This layered strategy would improve both sensitivity and specificity in challenging diagnostic settings. Additionally, we intend to explore uncertainty-aware modelling to quantify model confidence and help flag borderline predictions for radiologist review. Given the scarcity of labelled examples for ultra-small lesions (e.g., <5 mm), we will also incorporate active learning strategies that prioritise ambiguous or underrepresented cases for expert annotation, improving data efficiency.

## Data Availability

The original contributions presented in the study are included in the article/supplementary material, further inquiries can be directed to the corresponding author.

## Footnotes

1
https://www.med.upenn.edu/cbica/brats2021/

2
https://wiki.cancerimagingarchive.net/pages/viewpage.action?pageId=5309188

3
https://figshare.com/articles/dataset/brain_tumor_dataset/1512427

4
https://wiki.cancerimagingarchive.net/display/Public/LGG-1p19qDeletion#257890426456c02bce974ad7bc00dd80c274c914

## In memoriam

Álvaro J. García-Tejedor (deceased 1 January 2025). The authors gratefully acknowledge his valuable contributions to this work and attest that, to the best of their knowledge, he fulfilled the authorship criteria in accordance with Frontiers’ authorship policies before his passing.
